# Exploring the Relationship between Body Composition and Eating Behavior Using the Three Factor Eating Questionnaire (TFEQ) in Young New Zealand Women

**DOI:** 10.3390/nu8070386

**Published:** 2016-06-23

**Authors:** Rozanne Kruger, Jacqui G. De Bray, Kathryn L. Beck, Cathryn A. Conlon, Welma Stonehouse

**Affiliations:** 1 School of Food and Nutrition, College of Health, MIFST, Massey University, Auckland 0745, New Zealand; jacqui.debray@gmail.com (J.G.B.); k.l.beck@massey.ac.nz (K.L.B.); c.conlon@massey.ac.nz (C.A.C.); welma.stonehouse@csiro.au (W.S.); 2 CSIRO, Food and Nutrition, Adelaide 5001, Australia

**Keywords:** TFEQ, eating behavior, obesity, body fat percentage, disinhibition, restraint, hunger, women, New Zealand

## Abstract

Obesity is a leading cause of morbidity and mortality, yet is preventable. This study aimed to investigate associations between body mass index, body fat percentage and obesity-related eating behaviors. Women (*n* = 116; 18–44 years) were measured for height, weight and body fat using air displacement plethysmography (BodPod). Women completed the validated Three Factor Eating Questionnaire to assess their eating behaviors using Restraint, Disinhibition and Hunger eating factor categories and sub-categories. The eating behavior data were analyzed for associations with body mass index and body fat percentage, and comparisons across body mass index and body fat percentage categories (< vs. ≥25 kg/m^2^; < vs. ≥30%, respectively). Women had a mean (standard deviation) body mass index of 23.4 (3.5) kg/m^2^, and body fat percentage of 30.5 (7.6)%. Disinhibition was positively associated with both body mass index (*p* < 0.001) and body fat percentage (*p* < 0.001). Emotional Disinhibition was positively associated with body fat percentage (*p* < 0.028). Women with low Restraint and high Disinhibition had significantly higher body mass index and body fat percentage than women with high Restraint and low Disinhibition. Disinhibition seems likely to be an important contributor to obesity. Tailored intervention strategies focused on counteracting Disinhibition should be a key target area for managing weight/fat gain.

## 1. Introduction

Overweight and obesity are some of the leading risk factors for morbidity, with levels of obesity doubling globally since 1980 [1]. In many countries, including New Zealand (NZ), obesity is the leading modifiable risk factor for a number of diseases including type 2 diabetes, ischemic heart disease and stroke [2]. Life expectancy can be shortened by two to four years in obese individuals (30–35 kg/m^2^) and eight to ten years in extremely obese individuals (40–45 kg/m^2^) [3]. According to the NZ Health Survey published in 2015, 32% of women are classified as obese (>30 kg/m^2^). An estimated 147,000 (4%) of NZ adults are classified as extremely obese (≥40 kg/m^2^) and the highest rates are seen in young women aged 25 to 44 years [4]. These findings highlight the urgent need to address weight gain in healthy young women.

The causes of obesity are multi-faceted, and include psychological and behavioral factors. Previous research has shown that eating behavior is a strong predictor of weight gain in women [5,6]. By understanding the eating behaviors which are associated with a range of body weights or body fat percentages (BF %), we may be able to target behavioral interventions to prevent weight gain in young women.

Individuals vary in their eating behaviors with regard to food choices, eating food in excess of their needs and the subsequent effects on body weight (see review by French et al. [7]). Efforts made to assess eating behavior have led to the development of the Three Factor Eating Questionnaire (TFEQ) in the mid-1980s [8]. It is a widely-used and valid tool for assessing eating behaviors differentiating between normal weight or overweight and obese individuals [5,8,9,10,11], and has been validated in various ethnicities [12,13,14]. The TFEQ is a 51-item questionnaire which assesses three factors (and their sub-categories) [6,8,11] that refer to cognitions and behaviors associated with eating. These are: (1) cognitive dietary restraint (Restraint)—conscious control of food intake with concerns about body shape and weight; (2) disinhibition of control (Disinhibition)—overconsumption of food due to a variety of stimuli associated with a loss of control over food intake; and (3) susceptibility to hunger (Hunger)—food intake or eating in response to feelings and subjective perceptions of hunger [6,8,15,16,17].

The TFEQ has been used to investigate the relationship between the three eating behaviors and overweight/obesity [18,19,20,21,22,23]. Several studies have concluded that Restraint and Disinhibition scores were higher in women compared to men [6,15,24,25]. Stunkard and Messick [8] showed an interaction between Restraint and Disinhibition predicted body mass index (BMI), with a higher BMI associated with lower Restraint and higher Disinhibition scores. Disinhibition has been shown to be the strongest predictor of BMI, weight gain over time (as BMI) and the development of obesity [12,26,27,28,29]. Sub-categories of the TFEQ are now frequently used to improve our understanding of appetite control and body weight [15,27]. However, associations between eating behaviors, using the TFEQ categories and sub-categories, and BF % [6,30,31] have been investigated to a lesser extent. While BMI is a good general guide to identify overweight/obesity, it does not distinguish fat mass from fat free mass. For example, some individuals may have high levels of adiposity but a normal BMI [32].

Eating behaviors in relation to appetite response have been explored in a small group of ‘obesity susceptible’ and ‘obesity resistant’ NZ men and women using the TFEQ categories [33]. To our knowledge, no other studies have investigated the relationship between TFEQ scores and body composition in young NZ women. This study therefore aims to investigate the relationship between BMI and BF % and eating behaviors using the TFEQ.

## 2. Materials and Methods

### 2.1. Study Population and Procedure

Healthy, young women, aged between 18 and 44 years, were recruited (*n* = 116) from Auckland, NZ, as this group is at high risk of developing overweight and obesity. In addition, limiting the age range excluded the potential confounding effects of hormonal influences associated with menopause. Potential participants identified from the Human Nutrition Research Unit (HNRU) database were invited to take part in the study. An email advertisement was also sent to readers of the Healthy Food Guide magazine (a healthy eating magazine targeted at the general population). Exclusion criteria included any chronic disease, smoking, pregnancy, breastfeeding, or planning to become pregnant in the next six months.

Sample size was calculated using G*Power 3.13. To identify which of the three categories from the TFEQ were associated with BMI and BF % using multiple linear regression, a sample size of 77 was needed (80% power at a significance level of *p* < 0.05, medium effect size (f^2^ = 0.15)). For seven TFEQ sub-categories a total sample size of 103 was required.

Participants visited the HNRU at Massey University in Auckland from March to May 2009 for data collection. Participants completed a demographic questionnaire, the TFEQ and had anthropometric and body composition assessments done.

Written informed consent was obtained from all participants. Ethical approval was obtained from the Massey University Human Ethics Committee: (Southern A), Reference No 08/63.

### 2.2. Three Factor Eating Questionnaire

The 51-item TFEQ, consists of three eating behavior factors including Restraint (21 items), Disinhibition (16 items), and Hunger (14 items), and sub-categories providing further insight into each of the eating behaviors. Responses on the TFEQ are scored 0 or 1 and summed. Higher scores denote higher levels of restrained eating, disinhibited eating and predisposition to Hunger, respectively [8].

Restraint refers to the tendency of some individuals to restrict their food intake in order to control their body weight. Sub-categories include flexible and rigid control [15]. Flexible control has been described as a “more gradual approach to eating” [23], and refers to the ability of an individual to monitor their diet and employ Restraint where required to maintain their weight. Rigid control indicates an “all or nothing approach to eating, dieting and weight control” [21].

Disinhibition, or “counter-regulation”, is an overconsumption of food in response to a variety of stimuli, such as emotions or alcohol [8]. Disinhibition has been used to explain a loss of control in those with high Restraint scores. Sub-categories include habitual, emotional and Situational Disinhibition [16,34]. Habitual Disinhibition is susceptibility to overeat in response to daily life circumstances. Emotional Disinhibition occurs in response to emotional states such as anxiety and depression, whereas Situational Disinhibition is a tendency to overeat in response to environmental cues such as social occasions [28].

Susceptibility to Hunger (or perceived hunger) refers to food intake in response to feelings and perceptions of hunger [16,34]. Sub-categories relate to whether individuals tend to respond to internal (e.g., “I am usually so hungry that I eat more than three times a day”) or external (e.g., “being with someone who is eating often makes me hungry enough to eat also”) hunger cues [16].

### 2.3. Anthropometric and Body Composition Measurements

Height and body weight were determined using the International Society for the Advancement of Kinanthropometry protocols [35]. Quetelet’s BMI (kg/m^2^) was calculated from height (cm) and weight (kg).

Body composition analysis was conducted via air displacement plethysmography (2007A, Life Measurement Inc., Concord, CA, USA, software V4.2+ supplied by the manufacturer) following the thoracic gas volume method in the BodPod^®^ [30,31], using standardized procedures [36].

### 2.4. Statistical Analysis

All statistical analyses were completed using PASW Statistics 22 for Windows (SPSS, Inc., Chicago, IL, USA).

Tests of normality were completed on all data using visual inspection of histograms, the Shapiro-Wilk test and the Kolmogorov-Smirnov test. Normally distributed data were described using mean (SD), non-normally distributed data as median (25, 75 percentile) and categorical data using frequency summary statistics.

Multiple regression analysis (forced entry) was undertaken to identify the categories and sub-categories of the TEFQ associated with BMI and BF %. Assumptions for multicollinearity were met. 

Categories and sub-categories of the TFEQ data were compared according to categories of body weight/composition status (overweight and/or obese (OWOB) (BMI ≥25 vs. <25kg/m^2^), and High BF % (≥30 vs. <30%) independent of BMI). Differences between body composition categories were compared using Mann-Whitney tests. Cohen’s d effect size was calculated for any significant *p* values (*p* < 0.05). Using the recommendations of Lesdema et al. [25], low and high scorers within the sub-categories (Disinhibition (≤7 versus >7), Hunger (≤5 versus >5), and Restraint (≤7 versus >7) were identified and four sub-groups were formed (low Restraint-low Disinhibition; low Restraint-high Disinhibition; high Restraint-low Disinhibition; high-Restraint-high Disinhibition); comparisons between groups were performed using ANOVA and the Kruskall-Wallis test for normally and non-normally distributed data respectively. Where significant effects were found, post hoc-tests (Tukey and Mann-Whitney) were used to identify where the significant differences were. Two sided tests were used for all comparisons [37].

## 3. Results

### 3.1. Participants

Data from 116 NZ women, aged between 18 and 44 years were included in the analysis. The characteristics of the women are presented in Table 1. Median (25th, 75th percentile) scores for the TFEQ factors were 9 (6, 12) for cognitive restraint (high), 6 (4, 9) for disinhibition (moderate) and 5 (3, 7.8) for susceptibility to hunger (moderate) [25].

Table 2 outlines the main TFEQ factors namely Disinhibition, Hunger and Restraint, as predictors of BMI and BF %. The behavior scores explained approximately 18% and 12% of the BMI and BF % respectively, and Disinhibition was a significant predictor of both BMI and BF % (Table 2). For every point increase in Disinhibition score, BMI increased by 0.4 kg/m^2^ and 0.82% body fat. For the average woman in this study (1.66 m and 61.9 kg) this meant a 0.15 kg increase in body weight and an increase from 30.4% to 31.2% BF %. Neither Hunger nor Restraint was significantly associated with BMI.

### 3.2. TFEQ Sub-Categories’ Correlation to BMI and BF %

None of the sub-categories of TFEQ factors were found to be significant predictors of BMI. Emotional Disinhibition was the only sub-category of the TFEQ factors which significantly predicted BF %, with every point increase in Emotional Disinhibition increasing body fat by 1.59% (Table 3). Age was not a significant predictor nor did it change the final outcomes when added to these multiple regression models (data not shown).

### 3.3. Difference in TFEQ Sub-Categories Between Body Weight/Composition Groups

Overall, emotional and situational disinhibition were significantly different between OWOB and normal weight women, and women with normal versus high BF % (small to medium effect size) (Table 4). The overall Hunger score was significantly higher in OWOB versus normal weight women, but did not differ significantly between women with high versus lower BF %. There were no significant differences between groups in BMI and BF % categories for the sub-categories of Hunger or all categories of Restraint.

The four sub-groups based on the recommendations of Lesdema et al. [25], were compared using different combinations of high and low Restraint and Disinhibition scores (Table 5). Women in the low Restraint and high Disinhibition grouping had a significantly higher BMI and BF % than women in the high Restraint low Disinhibition group.

## 4. Discussion

The aim of this study was to investigate the relationships between BMI and BF % and obesity-related eating behaviors (cognitive dietary restraint, disinhibition and susceptibility to hunger) using the TFEQ in 116 young, healthy NZ women aged between 18 and 44 years. In addition to the major eating behaviors, the sub-categories of TFEQ behaviors were explored in relation to BMI and BF %.

Average scores for the three TFEQ sub-categories for the women in this study could be considered as modest to high. These results are comparable with several other studies conducted in young women; French women [25] and Canadian non-vegetarian women [31] had TFEQ scores similar to those of the women in our study for Restraint, Disinhibition and Hunger. In a NZ study investigating appetite responses of women, those classified as ‘obesity susceptible’ had similar TFEQ scores to our study [33]. Studies conducted in older women also showed very similar TFEQ scores [21,28].

Disinhibition was the characteristic most strongly associated with both BMI and BF %. Previous studies employing the TFEQ to compare behaviors between obese and non-obese individuals (based on BMI) have also found Disinhibition to be the TFEQ factor with the strongest association with BMI [18,21,23]. In congruence with this association, Disinhibition scores were significantly higher in OWOB versus normal weight women and in women with ≥30% BF versus <30% BF which is in agreement with previous research [10,12,19,21,38].

Emotional Disinhibition significantly predicted BF %, and was significantly higher in OWOB versus normal weight women and women with high versus low BF %. Although Situational Disinhibition did not predict BMI or BF % when controlling for other sub-categories of the TFEQ, Situational Disinhibition was higher in OWOB women and those with higher BF %. Habitual Disinhibition did not appear to be characteristic of those who were overweight or had a high BF %. Other studies have found both Emotional and Habitual Disinhibition to correlate with weight gain, whilst Situational Disinhibition showed no association [25,28].

In their original work on human eating behaviour, Stunkard and Messick [8] suggested that participants with high Disinhibition scores would benefit from behavioural management strategies, particularly with regards to emotional disinhibitors like anxiety or loneliness, and this may be particularly successful if addressed in a group support environment. The results of this study suggest Emotional Disinhibition was the most significant predictor of BF %, further supporting the suggestion that emotional disinhibitors should be a focus in obesity prevention programs. Löffler et al. [12] also reported in their German population study that emotional eating showed a weak positive association with BMI, thereby explaining that high levels of food intake with an emotional basis is related to excess weight status. In contrast, other studies have shown that Habitual Disinhibition may be the most important quantitative correlate of weight gain and high BMI, with Emotional Disinhibition playing a secondary role [25,28]. According to Hays and Roberts [28], this may be due to the current environment which allows access to highly palatable food and a higher number of eating opportunities. Other studies have found that Situational Disinhibition was the least important in weight gain, in contrast to the perception that social occasions or environmental cues drives eating behavior [28]. The current study conducted in a sample of healthy young women suggests Emotional Disinhibition should be the focus of tailored prevention strategies.

Restraint was not associated with BMI or BF % in the current study. Other studies have shown mixed results; either failing to demonstrate a strong relationship similar to our results [6,15,19,21,30,39,40], or demonstrating a significant relationship [25,31,38]. However, it is well known that rigid and flexible control are oppositely related to obesity status (high BMI and low BMI respectively), and together they may show no association with BMI, hence it has been suggested to focus analysis on the sub-scales of the Restraint measures to identify appropriate treatment strategies [6,15]. However, there were no significant differences in body composition categories for sub-categories of Restraint in this study.

Although Hunger was not significantly associated with BMI or BF %, Hunger scores were significantly greater in OWOB women compared to their normal weight counterparts (although the effect size was small). Other studies have reported significant positive associations between Hunger, and/or External Hunger and body weight or BMI measures [18,21]. Behaviors related to Hunger may be more important as a person becomes obese due to metabolic dysregulation of satiety signals [18].

It has been suggested that the combination of high Restraint and low Disinhibition may be an important predictor of successful weight maintenance when using a dietary approach [19]. Women in our study with these characteristics (High-Restraint-Low-Disinhibition) had the lowest BMI scores (mean: 22.3 kg/m^2^), and also the least body fat (mean: 28.1 BF %), both of which are within normal parameters for body composition. On the other hand, women with Low-Restraint-High-Disinhibition characteristics tended to have the highest BMI scores (mean: 26.2 kg/m^2^) and the highest amount of body fat (mean: 36.5 BF %). These findings are in line with others suggesting an interaction between Restraint and Disinhibition domains of eating behavior [12,41]. Borg et al. [19] found that focused changes on the short term were achievable for weight change in individuals with these characteristics, but the ability to maintain weight changes was lacking. Successful dietary and weight maintenance is more likely to be achieved if behavior is characterized by high dietary Restraint and low Disinhibition. This is achievable through reinforcement of positive self-monitoring behavior and self-efficacy [19]. Furthermore, it has been suggested that success with weight management is related to high Rigid Control, whilst increasing weight is associated with low Flexible control [15]. Higher Flexible Control could be achieved by balancing energy intake, healthy eating, implementing strategies to control stimuli in the environment and coping with External Hunger [8].

This study had sufficient power to detect significant associations between TFEQ behaviors, including their sub-categories and BMI and BF %. Another strength of this study is that BMI and BF % were measured whereas many other studies use self-reported BMI (which may be lower than measured data) [25,28] or did not measure BF % [19,21]. Limitations include the fact that this study was conducted in women only, and that the majority of participants were European and of a healthy weight, meaning the study is limited in its generalizability to men, cultures other than NZ Europeans as well as to obese groups. Furthermore the sample was a small volunteer sample interested in health, meaning the results are unable to be applied to the general population. The cross-sectional study design means causality cannot be inferred.

## 5. Conclusions

In order to stem escalating rates of obesity in the Western world prevention strategies need to improve. By addressing the behaviors involved in the pathogenesis of obesity we may improve the success of preventative interventions. Of the TFEQ behaviors, we identified Disinhibition as being the strongest predictor of both BMI and BF % in women of healthy body weight. Disinhibition, particularly Emotional Disinhibition, may be an important factor in weight gain as it predicts BF % as well as being associated with overweight status. Therefore intervention strategies which educate young women with higher than normal BMI or BF % on strategies to counteract Disinhibition are imperative.

## Figures and Tables

**Table 1:** Participant characteristics.

Characteristic	Median (25th, 75th Percentile)
Age (years)	34 (27, 40)
BMI (kg/m^2^)	23.4 (3.5) ^1^
Body fat (%)	30.5 (7.6) ^1^
Ethnic group n (%):	
*European*	87 (83.6)
*Maori*	6 (5.2)
*Pacific Island*	0 (0)
*Asian*	8 (6.9)
*Other*	5 (4.3)
TFEQ factors:	
Restraint (range 0–21)	9 (6, 12)
Flexible	3 (2, 4)
Rigid	3 (2, 4)
Hunger (range 0–14)	5 (3, 7.8)
External	2 (1, 3)
Internal	2 (1, 3)
Disinhibition (range 0–16)	6 (4, 9)
Situational	3 (1.3, 4)
Habitual	1 (0, 2)
Emotional	1 (0, 2)

^1^ Mean (SD). Three Factor Eating Questionnaire (TFEQ).

**Table 2:** Multiple Linear Regression of TFEQ factors correlation to body mass index (BMI) and body fat percentage (BF %).

**Model ^a^ for BMI**	**Β**	**Std Error β**	**95% CI β**	**Std’ised β**	***p*** **Value**
Intercept	20.88	0.97	18.95–22.80		<0.001
Disinhibition	0.40	0.10	0.21–0.59	0.40	<0.001
Hunger	0.11	0.12	−0.12–0.34	0.09	0.36
Restraint	−0.07	0.07	−0.20–0.07	−0.08	0.34
^a^ = *F* = 9.318 (3, 115), adjusted *R*^2^ = 0.178					
**Model ^a^ for BF %**	**Β**	**Std Error β**	**95% CI β**	**Std’ised β**	***p*** **Value**
Intercept	27.88	2.17	23.58–32.18		<0.001
Disinhibition	0.82	0.21	0.40–1.24	0.38	<0.001
Hunger	−0.09	0.26	−0.60–0.42	−0.04	0.72
Restraint	−0.25	0.15	−0.55–0.06	−0.14	0.11
^a^ = *F* = 6.380 (3, 115), adjusted *R*^2^ = 0.123					

**Table 3:** Multiple Linear Regression of TFEQ sub-category factors’ correlation to BMI and BF %.

**Model ^a^ for BMI**	**Β**	**Std Error β**	**95% CI β**	**Std’ised β**	***p*-Value**
Intercept	22.27	1	20.29–24.26		<0.001
Flexible control	−0.41	0.22	−0.85–0.02	−0.20	0.064
Rigid control	0.10	0.23	−0.35–0.55	0.05	0.661
Situational Disinhibition	0.32	0.28	−0.23–0.87	0.13	0.250
Habitual Disinhibition	0.45	0.28	−0.10–0.99	0.18	0.108
Emotional Disinhibition	0.61	0.32	−0.03–1.25	0.19	0.061
External Hunger	−0.10	0.29	−0.68–0.48	−0.04	0.722
Internal Hunger	0.15	0.23	−0.31–0.60	0.07	0.526
^a^ = *F* = 4.180 (7, 115), adjusted *R*^2^ = 0.162					
**Model^a^ for BF %**	**Β**	**Std Error β**	**95% CI β**	**Std’ised β**	***p*-Value**
Intercept	30.26	2.22	25.87–34.65		<0.001
Flexible Control	−0.86	0.49	−1.83–0.10	−0.20	0.079
Rigid Control	0.02	0.50	−0.97–1.01	0.00	0.970
Situational Disinhibition	0.41	0.62	−0.81–1.63	0.08	0.506
Habitual Disinhibition	0.68	0.61	−0.53–1.89	0.13	0.266
Emotional Disinhibition	1.59	0.71	0.18–3.00	0.23	0.028
External Hunger	0.15	0.65	−1.14–1.43	0.03	0.822
Internal Hunger	−0.46	0.51	−1.48–0.55	−0.10	0.368
^a^ = *F* = 3.127 (7, 115), adjusted *R*^2^ = 0.115					

**Table 4:** Exploration of Disinhibition and sub-categories in relation to BMI and BF % sub-groups.

	BMI			BF %		
	Normal Weight (<25 kg/m^2^)	OWOB (≥25 kg/m^2^)	Difference between Groups (*p* Value)	Normal BF% (<30%)	High BF % (≥30%)	Difference between Groups (*p* Value)
	*n* = 84	*n* = 32		*n* = 60	*n* = 56	
**DISINHIBITION**	**5 (3, 7.8)**	**8 (5.3, 11)**	**<0.001, *r* = 0.35**	**5 (3, 7.8)**	**7 (4.3, 11)**	**0.003, *r* = 0.28**
Situational Disinhibition	2 (1, 3)	3 (2, 4)	0.006, *r* = 0.25	2 (1, 3)	3 (2, 4)	0.003, *r* = 0.28
Habitual Disinhibition	1 (0, 2)	1 (0, 3.8)	0.071	1 (0, 2)	1 (0, 2)	0.336
Emotional Disinhibition	1 (0, 2)	2 (0.3, 3)	0.003, *r* = 0.28	1 (0, 2)	1 (0, 2)	0.023, *r* = 0.21
**HUNGER**	**4 (3, 7)**	**6.5 (3.3, 9)**	**0.021, *r* = 0.21**	**5 (3, 7.8)**	**5 (3, 7.8)**	**0.872**
External Hunger	2 (1, 3)	2 (1, 4)	0.058	2 (1, 3)	2 (1, 3)	0.586
Internal Hunger	2 (1, 3)	3 (1, 4)	0.063	2 (1, 3.8)	2 (1, 3)	0.554
**RESTRAINT**	**9 (6.3, 12)**	**9 (5.3, 12.8)**	**0.845**	**9 (7, 12)**	**8.5 (5, 12)**	**0.128**
Flexible Control	3 (2, 4)	2.5 (1, 4)	0.266	3 (2, 4)	2 (1, 4)	0.096
Rigid Control	3 (2, 4)	3 (2, 4)	0.219	3 (2, 4)	2.5 (2, 4)	0.146

Presented as median (25th, 75th percentiles); difference between groups (Mann-Whitney test), OWOB = overweight/obese. *r* = 0.1 (small effect), *r* = 0.3 (medium effect).

**Table 5:** Participant characteristics according to the level of Restraint and Disinhibition.

	Low Disinhibition Low Restraint (*n* = 27)	Low Disinhibition High Restraint (*n* = 49)	High Disinhibition Low Restraint (*n* = 16)	High Disinhibition High Restraint (*n* = 24)
Age ^1^	35 (28, 39)	31 (26.5, 40)	33.5 (26.3, 41.5)	33 (25, 40.5)
BMI ^2^	22.9 (2.7) ^a^	22.3 (2.8) ^b,c^	26.2 (5.1) ^a,b^	24.5 (3.4) ^c^
BF % ^2^	30.2 (6.3) ^a^	28.1 (7.0) ^b^	36.5 (8.9) ^a,b^	31.6 (7.2)

^1^ Medians (25th, 75th percentiles); Kruskal-Wallis; ^2^ Mean (SD); One way ANOVA; Values with similar superscript letters are significantly different (*p* < 0.05).

## Abbreviations

The following abbreviations are used in this manuscript:

BMI: Body Mass Index
BF %: Body Fat Percentage
HNRU: Human Nutrition Research Unit
NZ: New Zealand
OWOB: Overweight and/or obese
TFEQ: Three Factor Eating Questionnaire
